# Dipotassium Glycyrrhizate Improves Intestinal Mucosal Healing by Modulating Extracellular Matrix Remodeling Genes and Restoring Epithelial Barrier Functions

**DOI:** 10.3389/fimmu.2019.00939

**Published:** 2019-04-26

**Authors:** Laura Stronati, Francesca Palone, Anna Negroni, Eleonora Colantoni, Anna Barbara Mancuso, Salvatore Cucchiara, Vincenzo Cesi, Sara Isoldi, Roberta Vitali

**Affiliations:** ^1^Department of Molecular Medicine, Sapienza University of Rome, Rome, Italy; ^2^Pediatric Gastroenterology and Liver Unit, Department of Pediatrics, Sapienza University of Rome, Rome, Italy; ^3^Division of Health Protection Technologies, Territorial and Production Systems Sustainability Department, ENEA, Rome, Italy

**Keywords:** dipotassium glycyrrhizate, mucosal healing, DSS-induced colitis, plaur, VTN

## Abstract

Gut mucosal healing (MH) is considered a key therapeutic target and prognostic parameter in the management of inflammatory bowel disease (IBD). The dipotassium glycyrrhizate (DPG), a salt of the glycoconjugated triterpene glycyrrhizin, has been shown to inhibit the High Mobility Group Box 1 (HMGB1) protein, an allarmin strongly implicated in the pathogenesis of most inflammatory and auto-immune disorders. Here we discuss new insights on how DPG acts on MH comparing the acute phase and the recovery phase from experimental colitis in mice. We found that DPG strongly accelerates MH by differently regulating pro-inflammatory (CXCL1, CXCL3, CXCL5, PTGS2, IL-1β, IL-6, CCL12, CCL7) and wound healing (COL3A1, MMP9, VTN, PLAUR, SERPINE, CSF3, FGF2, FGF7, PLAT, TIMP1) genes as observed only during the recovery phase of colitis. Relevant issue is the identification of extracellular matrix (ECM) remodeling genes, VTN, and PLAUR, as crucial genes to achieve MH during DPG treatment. Furthermore, a noticeable recovery of intestinal epithelial barrier structural organization, wound repair ability, and functionality is observed in two human colorectal adenocarcinoma cell lines exposed to DPG during inflammation. Thus, our study identifies DPG as a potent tool for controlling intestinal inflammation and improving MH.

## Introduction

Inflammatory bowel diseases (IBD), are complex disorders of the gastrointestinal (GI) tract whose major phenotypes are Crohn's disease (CD), and ulcerative colitis (UC). IBD are featured by chronic inflammation which may result in irreversible mucosal damage, disability, and heightened incidence of colitis-associated neoplasias (1, 2).

Recent studies have identified gut mucosal healing (MH) as key therapeutic target and prognostic parameter in the management of IBD. Indeed, current and novel treatment options are considered and selected by their efficacy in inducing MH (3–5). The structural basis of MH is the restitution of gut epithelium that prevents translocation of commensal bacteria into the mucosa and subsequent immune-stimulation (6, 7). Achieving MH also prompts better long-term results, such as less hospitalization and surgery as well as better quality of life, with a critical impact on the natural history of the disease (8).

Intestinal cells undergoing severe inflammation release cytokines and other factors, including damage-associated molecular pattern molecules (DAMPs), that, in turn, activate inflammatory signaling pathways such as NF-κB. High Mobility Group Box-1 (HMGB1) is a DAMP prototype that normally resides in the nucleus, where it functions as a structural co-factor critical for proper transcriptional regulation in somatic cells. However, under appropriate signal stimulation, HMGB1 is released into the extracellular milieu and activates the immune system promoting inflammation (9, 10). Indeed, HMGB1 has been implicated in several inflammatory and auto-immune disorders, such as sepsis syndromes, rheumatoid arthritis, systemic lupus erythematosus and ankylosing spondylitis (11). Thus, inhibition of HMGB1 might represent a promising clinical approach for treating tissue inflammation (12, 13).

Recently, our group showed that HMGB1 is highly expressed in the inflamed intestinal tissues of CD and UC patients (14–16). Moreover, we showed that the dipotassium glycyrrhizate (DPG), a salt of the glycoconjugated triterpene glycyrrhizin (GL), a major active constituent of *Glycyrrhiza glabra* root, significantly improves the experimental colitis in mice by inhibiting HMGB1 (17).

The aims of the present study were: (1) to identify genes involved in MH pathways modulated by DPG in mice with DSS-induced acute colitis; (2) to investigate *in vitro* the outcome of DPG on MH by analyzing the impairment of epithelial cell migration, morphology, and functionality during inflammation; (3) to determine potential different effects of DPG on MH genes during a recovery phase following the DSS-induced colitis in mice; (4) to identify *in vitro* the DPG-affected genes that are crucial for efficient MH.

## Materials and Methods

### Animals

C57BL/6 female mice (8–9 weeks of age; Harlan Laboratories, Udine, Italy) were housed in collective cages at 22/21°C under a 12-h light/dark cycle and with food and water provided *ad libitum*.

#### Induction of Acute Colitis

Mice were given 3% dextran sodium sulfate (DSS, molecular mass, 36,000–50,000 Da, MP Biomedicals, Santa Ana, CA), dissolved in autoclaved drinking water, for 7 days and then sacrificed. Mice, 8 for group, were randomly divided into three groups: (1) control group received regular drinking water; (2) mice treated with 3% (w/v) DSS; (3) mice treated with 3% DSS and 8 mg/kg/day DPG (DMG Italia Srl, Pomezia, italy), diluted in Phosphate Buffered Saline (PBS), administered by oral gavage.

#### Recovery After DSS-Induced Colitis

Mice, were given 3% DSS in drinking water for 5 days followed by 9 days of regular water or 8 mg/kg/day DPG, administered by oral gavage; mice were divided into the following groups (5 animals per group): (1) mice treated with DSS for 5 days and then sacrificed; (2) mice treated with DSS for 5 days, then DSS was removed and animals were treated with a vehicle (PBS) at 6, 24 (day 6), 72 (day 8), 144 (day 11), 216 (day 14) hours and then sacrificed; (3) mice treated with DSS for 5 days, then DSS was removed and animals were treated with 8 mg/kg/day DPG at 6, 24 (day 6), 72 (day 8), 144 (day 11), 216 (day 14) hours and then sacrificed.

#### Assessment of DSS-Induced Colitis and Histological Score

Animals were daily examined and the clinical score (CS) was assessed by evaluating stool consistency (0 for normal stool, 1 for moist/sticky stool, 2 for soft stool, 3 for diarrhea), presence of blood in stools (0 for no blood, 1 for evidence of blood in stools or around anus, and 2 for severe bleeding) and general appearance of the animal (0 was assigned if normal, 1 for ruffled fur or altered gait, 2 for lethargic or moribund), according to Maxwell et al. (18). Mice were daily weighed, and the percentage of weight loss was calculated in relation to the starting weight. The 7th day, animals were euthanized, colon removed and examined for weight and length. Distal colonic specimens were frozen in liquid nitrogen and stored at −80°C for further analyses. For histological analysis, samples were fixed immediately in a 10% (w/v) formalin solution and embedded in paraffin, sectioned (4 μm thickness), mounted on glass slides. Slices were stained using standard hematoxylin and eosin (H&E) techniques. Samples were analyzed by light microscopy and scored according to Maxwell et al. (18).

### Ethic Statement

Experimental procedures were previously approved by the Ministry of Health and the study was carried out in accordance with the Italian regulations on animal welfare. The protocol was approved by the Committee on the Ethics of Animal Experiments of the Italian National Agency for New Technology, Energy and Sustainable Economic Development (ENEA-Permit Number: 1175/2016-PR).

### RT2 Profiler PCR Array on Genes of Wound Healing Pathway

Total RNA was isolated from mouse colonic tissues using the RNeasy Microarray tissue kit (Qiagen), and 1 μg of total RNA was reverse transcribed by the RT2 First Strand Kit (Qiagen). Samples were analyzed by a RT2 Profiler PCR array (Qiagen) to evaluate the expression levels of a panel of 84 genes central to MH response. Gene list: *Extracellular Matrix (ECM) and Cell Adhesion Molecules:* ECM Structural Constituents: COL14A1, COL1A1, COL1A2, COL3A1, COL4A1, COL4A3, COL5A2, VTN; ECM Remodeling Enzymes: Cathepsin G, Cathepsin K, Cathepsin V, F3 Coagulation Factor III, F13A1, FGA, MMP1, MMP2, MMP7, MMP9, PLAT, PLAU, PLAUR, PLG, PAI-1 (SERPINE), TIMP1; Cell Adhesion Molecules: CDH1, ITGA1, ITGA2, ITGA3, ITGA4, ITGA5, ITGA6, ITGAV, ITGB1, ITGB3, ITGB5, ITGB6; Cytoskeleton Regulators: RAC1, ACTA2 (α-SMA), ACTC1, TAGLN; *Inflammatory Cytokines and Chemokines:* C-C motif chemokine 7, CCL12 (MCP-1), CD40LG (TNFSF5), CXCL1, CXCL11, CXCL2, IFNG, IL-10, IL-1B, IL-2, IL-4, IL-6, MIP-2BETA (CXCL3); *Growth Factors:* CTGF, ANGPT1, GM-CSF (CSF2), CSF3 (GCSF), FGF2, FGF7, FGF10, HGF, IGF1, MIF, TGFA, TGFB1, TNF, PDGFA, VEGFA; *Signal Transduction:* TGFβ Signaling: TGFB1, STAT3; WNT Signaling: CTNNB1, WISP1; Kinases: ERK2, MAPK3, PTEN; Cell Surface Receptors: EGFR, IL6ST (GP130); *Other Signal Transduction Genes*: PTGS2 (COX2); A threshold of 3.5 times was chosen.

The normalization and all the data analysis were performed according to the manufacturer's instructions using their web-based software package: https://dataanalysis.qiagen.com/pcr/arrayanalysis. For the normalization it uses the average of five housekeeping genes: Actb, B2m, Gapdh, Gusb, Hsp90ab1. In order to identify false positives a statistical assessment of the False Discovery Rate were carried out by Benjamini–Hochberg procedure with critical value of 0.05 (http://www.biostathandbook.com/multiplecomparisons.html).

### Cell Lines

Caco2 and HT29 (HTB38, CL.19A) (human colorectal adenocarcinoma cell lines) were purchased from American Type Culture Collection (ATCC, Rockville, MA, USA). Caco2 and HT29 were maintained at 37°C, 5% CO_2_, in Dulbecco's minimum essential medium (DMEM, Gibco, Life Technologies, Carlsbad, CA, USA) and McCOY's 5A medium (Gibco), respectively, supplemented with 10% inactivated fetal bovine serum (FBS Eu Approved, Euroclone, Milan, Italy), 2 mM L-Glutamine, 100 U/ml penicillin and 100 μg/ml streptomycin (Euroclone). Caco2 were differentiated in culture medium for 16 days.

### Real Time-PCR

Total RNA was isolated from mouse colonic tissues using the mini RNeasy kit (Qiagen), and 1 μg of total RNA was reverse transcribed by IScriptTM cDNA Synthesis Kit (BioRad, Hercules). The RT-PCR amplifications were obtained by a BioRad CFX96 TouchTM Real-Time PCR Detection System using SsoAdvanced Universal SYBR Green super Mix (BioRad).

The primers used were summarize in Table 1. The expression level of each mRNA was assessed using the standard curve method and *GADPH* was used for normalization.

**Table 1:** List of murine primers used in RealTime PCR.

**Gene**	**Forward primer sequence 5^**′**^ → 3^**′**^**	**Reverse primer sequence 5^**′**^ → 3^**′**^**
IL-1β	GAGGCAGTATCACTCAATG	CGTTGCTTGGTTCTCCTTGT
Il-6	CAAGTCGGAGGCTTAATTACACATG	AGAAAAGAGTTGTGCAATGGCA
CCL12	GTTGGCTCAGCCAGATGAA	AGGCTACTCATTGGGATCATCTTG
CXCL3	GAAGATTACTGAAGAGCGGCAAGTC	AATGCAGGTCCTTCATCATGGT
CXCL5	CGTAACTCCAAAAATTAATCCCAAA	CGAGTGCATTCCGCTTAGCT
CCL7	CCTGGGAAGCTGTTATCTTCAA	AGGCTTTGGAGTTGGGGTTT
CXCL1	ACCGAAGTCATACCCACACTC	CTCCGTTACTGGGGGACACC
COL3A1	GCCCACAGCCTTCTACAC	CCAGGGTCACCATTTCTC
VTN	CCCCTGAGGCCCTTTTTCATA	CAAAGCTCGGTCACACTGACA
CSF3	GTATAAAGGCCCCCTGGAGCTG	TGCAGGGCCATTAGCTTCAT3
FGF2	GGAGGGCTGCTGGCTTCTAA	CCAGTTCGTTTCAGTGCCACATAC
FGF7	GAACAAAAGTCAAGGAGCAACC	GTCATGGGCCTCCTCCTATT
MMP9	TGTCTGGAGATTCGACTTGAAGTC	TGAGTTCCAGGGCACACCA
TIMP1	CTGGCATCTGGCATCCTCTT	TAGCCCTTATGACCAGGTCCG
PLAT	AGATGAGCCAACGCAGACAA	GTTGGTTGGCTGCAACTTGC
PLAUR	GTGGCCCAGTTCTGGATCTT	GATGAGAGACGCCTCTTCGG
SERPINE1	ACTGCAAAAGGTCAGGATCG	ACAAAGGCTGTGGAGGAAGA
PTGS2	AAGTGCGATTGTACCCGGAC	GTGCACTGTGTTTGGAGTGG
TNF	CAGACCCTCACACTCAGATCATCTT	TCGTAGCAAACCACCAAGTGG
IL-10	AACAAAGGACCAGCTGGACAAC	GGCAACCCAAGTAACCCTTAAA
GAPDH	AACTTTGGCATTGTGGAAGG	CACATTGGGGGTAGGAACAC

### Immunoblot Analysis

Mouse colonic tissues were suspended in ice-cold lysis buffer (50 mM Tris (pH 7.4), 5 mM EDTA, 250 mM NaCl, 0.1% Triton X-100, 1 mM phenylmethylsulfonyl fluoride, 5 mg/ml aprotinin, 5 mg/ml leupeptin, and 1 mM sodium orthovanadate (Sigma), homogenized and incubated in ice for 30 min. Samples were centrifuged at 14,000 r.p.m. for 10 min., supernatants collected and analyzed by western blot.

Ten microgram of total proteins, were fractionated by sodium dodecyl sulfate-polyacrylamide gel electrophoresis to detect selected MH proteins. Proteins were transferred in polyvinylidene fluoride membrane (Bio-Rad) and blocked with TBS-T (Tris-buffered saline with Tween-20) containing 5% non-fat dry milk. Anti-MMP9 (1:1,000; NovusBio), anti-VTN (1:1,000; NovusBio), anti-COL3A1 (1:1,000; ThermoScientific), anti-TIMP1 (1:1,000; Bioss), anti-PLAUR (1:1,000; Origene), and anti-β-actin (1:5,000; Sigma) antibodies were diluted in TBS-T containing 3% non-fat dry milk and incubated overnight at 4°C. Membranes were washed in TBS-T, incubated for 1 h with horseradish peroxidase-conjugated secondary antibody (Santa Cruz Biotechnology Inc.,), washed in TBS-T, and developed with ECL-Plus (GE Healthcare, Life Science). Densitometrical analyses of the blots were performed using the Software ImageQuant (GE Healthcare, Life Science).

### Wound Healing Assay

Wound Healing Assay was assessed by scratch test as previously described (19). HT29 cells were cultured in 6-well plated at a density of 2 × 10^5^ cells/ml until confluence reached 90%. A straight-line wound was made using a 10-ul pipette tip. Cell debris and smoothed the edge of the straight-line wound were removed by a wash with PBS and cells were then maintained in a medium with a reduced percentage of FBS (1%). Then cells were exposed to cytomix [TNFα (100 ng/ml) and INFγ (250 ng/ml), Peprotech, Rocky Hill, USA] or to B-box (10 μg/ml) (HMGBiotech, Milan, Italy) in presence or absence of DPG (300 μM) or anti-HMGB1 antibody (Sigma) for 48 h. In a second set of experiments, cells were treated with cytomix for 24 h and then were exposed to DPG (300 μM, Sigma), anti-VTN (1:1,000) and anti-PLAUR (1:1,000), alone or in combination, for 24 h. In both case, cells migrated into the wounded area were visualized at 0, 6, 24, and 48 h by Hematoxylin and Eosin staining. Images of each condition were acquired at a magnification of 10X. Cellular density related to a fixed wounded area (1 mm^2^) was measured after 48 h using ImageJ software (available in the public domain at www.nih.gov; National Institutes of Health [NIH], Bethesda, MD, USA) (10 acquisition for each experimental point). The experiment was replicated three times.

### Immunofluorescence

Cells exposed to cytomix or co-exposed to cytomix and DPG (300 μM) were grown at confluence on a microscope glass slide for 24 h. Then, cells were fixed in formalin 4% in PBS for 10 min. Cells were washed in PBS, permeabilized by incubation in PBS 0.1% Triton X-100 (Sigma) for 10 min and then blocked in PBS 1% BSA for 30 min. For ZO-1 (Tight junction protein-1) staining samples were incubated with anti-ZO-1 (1:100, BD Trasduction Laboratories) for 1 h and then with the secondary anti-mouse antibody AlexaFluor 488 (Molecular Probes, Eugene, OR, USA) for 30 min. F-actin was stained with Alexa Fluor 488-conjugated Phalloidin (1:50, Molecular Probes) according to manufacturer's instructions. 4′,6-diamidino-2-phenylindole (DAPI) was added for nuclei counterstaining. Quantification of ZO-1 and F-actin fluorescent signal relative to DAPI was performed using Image J software.

### Trans-epithelial Electrical Resistance (TEER) Measurements

Caco2 cells were seeded at a density of 1.0 × 10^5^ cells/well on ThinCert™ Cell Culture Inserts (24 well, 0.4 μm pore size, Greiner) and were differentiated for 16 days. Cellular TEERs were measured with an electrical resistance system, EVOM2, Epithelial Volt/Ohm Meter for TEER (EVOM2-TEER, WPI). When the cells reached a stable TEER readings >2,000 øcm^2^, cytomix or co-treatment with cytomix and DPG (300 μM) were supplied to apical and basal compartments of transwell chambers for 96 h. In a second set of experiments, cells were treated with cytomix for 24 h and TEER value was measured (t0); Cells were then exposed to DPG 300 μM (SIGMA), anti-VTN (1:1,000) and anti-PLAUR (1:1,000), alone or in combination for 48 h. In both case, TEER measurements were daily acquired. Results were expressed as percentage relative to the ratio between initial and TEER value at different time points.

### Statistics

All statistical analyses were performed with GraphPad InStat software (GraphPad Software, San Diego, CA, USA). The Kolmogorov–Smirnov test was used to assess whether data were sampled from populations following the gaussian distribution. Comparison among groups was performed using the Kruskal-Wallis Test with Dunn's Multiple Comparisons *post-test*.

For *in vitro* studies, all experiments were repeated three times and data were given as mean ± standard deviation (SD). Comparisons among groups was performed by Mann–Whitney test. Differences were noted as significant ^*^ *p* < 0.05, ^**^ *p* < 0.01, ^***^ *p* < 0.001.

## Results

### DPG Down-Regulates the Expression Levels of MH Genes Altered by Inflammation in Mice With Experimental Acute Colitis

**Figure 1:**
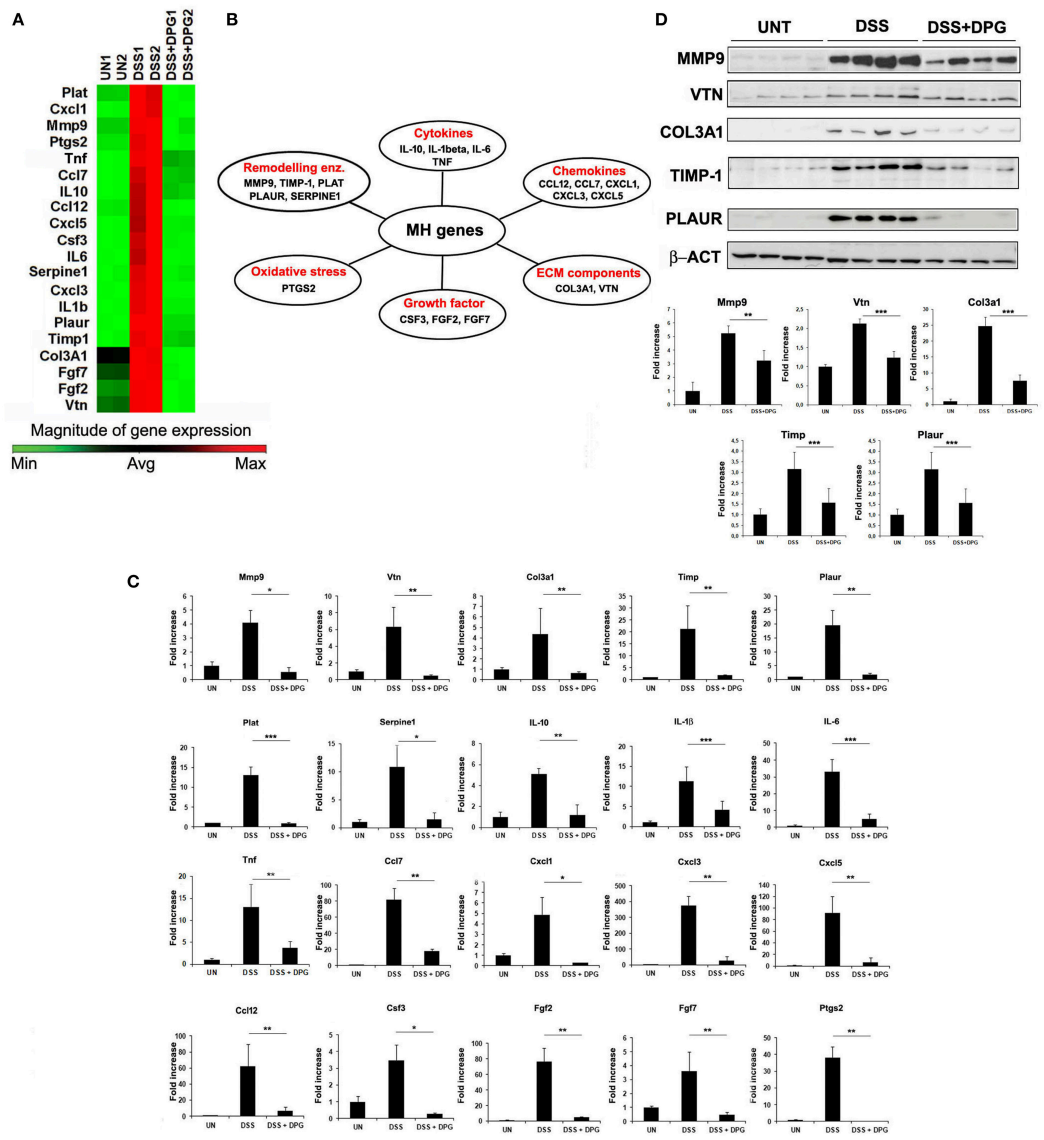
Identification and validation of genes, involved in MH pathways, modulated by DPG in inflamed tissues of mice with experimental acute colitis. **(A)** Genes activated by DSS and inactivated by DPG treatment *in vivo*, identified by PCR-Array, and **(B)** their classification in functional groups. **(C)** mRNA expression of all identified genes, modulated by DPG in mice with DSS acute colitis, were validate by quantitative real-time PCR. Statistical analysis: Kruskal-Wallis Test with Dunn's Multiple Comparisons *post-Test*. **(D)** Protein expression of some identified genes was confirmed by western blot, using 8 animals per group. Statistical analysis: Kruskal-Wallis Test with Dunn's Multiple Comparisons *post-Test*. *p < 0.05, **p < 0.01, ***p < 0.001. PCR-Array was repeated in triplicate using a pool of RNA obtained from 5 animals per group. Real-time PCR values are mean ± St. dev. of 8 animals per experimental group and data represent the target gene expression normalized to the reference gene. UN, untreated animals; DSS, dextran sulfate sodium; DPG, dipotassium glycyrrhizate.

We used an array of 84 genes of wound healing pathway to identify those more influenced by DPG in the colonic tissue of mice with acute colitis. C57BL/6 mice were randomly divided into three groups: control group received regular drinking water, mice treated with 3% DSS to induce colitis and mice treated with 3% DSS and 8 mg/kg/day DPG, daily administered by oral gavage. After 7 days, mice were sacrificed and the colon removed. RNA obtained from colon tissue of controls, DSS-induced colitis and mice co-treated with DSS and DPG, were analyzed by a PCR array. Results showed that inflammation induced by DSS caused a strong up-regulation of 24 out of 84 genes (*p* < 0.05), of which, 20 were significantly down-regulated by DPG administration (Figure 1A). Statistical assessment of the False Discovery Rate demonstrated that all identified modulation was significant (Supplementary Material 1). The 20 genes, down-regulated by DPG, were classified into the following functional groups: (1) *cytokines* (IL-10, IL-1β, IL-6, TNF); (2) *chemokines* (CCL12, CCL7, CXCL1, CXCL3, CXCL5); (3) *extracellular matrix components* (COL3A1, VTN); (4) *growth factors* (CSF3, FGF2, FGF7); (5) *extracellular matrix remodeling enzymes* (MMP9, TIMP1, PLAT, PLAUR, SERPINE1); (6) *oxidative stress* (PTGS2) (Figure 1B). The effect of DPG on mRNA expression of this gene set was then confirmed by RealTime-PCR (*p* < 0.001: IL-1beta, IL-6, PLAT; *p* < 0.01: IL-10, TNF, CXCL3, CXCL5, CCL12, CCL7, COL3A1, VTN, FGF7, FGF2, TIMP1, PLAUR, PTGS2; *p* < 0.05: CXCL1, MMP9, SERPINE1, CSF3) (Figure 1C). Moreover, a subset of genes (MMP9, VTN, COL3A1, TIMP1, and PLAUR), chosen among those more directly involved in the tissue remodeling process, were also analyzed by western blot. Results again showed that the expression levels of all genes previously altered by the DSS treatment was reduced by DPG (MMP9: *p* < 0.01; VTN, COL3A1, TIMP-1, PLAUR: *p* < 0.001) (Figure 1D). Complete list of 84 mucosal healing pathway genes analyzed was reported in Table 2. Moreover, data set of the microarray carried out are available on Gene Expression Omnibus platform (GEO) with accession number GSE127953 and provided as Supplementary Material 1.

**Table 2:** List of the mucosal healing pathway genes analyzed.

**Gene**	**Fold regulation comparing DSS vs. UN**	**Fold regulation comparing DSS+DPG vs. DSS**	
Cxcl3	1083.85	−165.97	
Il6	425.18	−102.17	
Csf3	335.14	−55.9	
Il1b	204.88	−37.48	
Il10	60.91	−9.35	
Ifng	49.13	−2.87	^*^Excluded DSS+DPG vs. DSS < 3.5 folds
Cxcl5	44.38	−21.33	
Cxcl1	28.68	−37.57	
Timp1	26.09	−8.5	
Ccl7	25.61	−7.15	
Tnf	17.09	−5.43	
Plaur	14.61	−8.78	
Ptgs2	13.04	−30.87	
Serpine1	10.55	−9.77	
Vtn	8.43	−40.55	
Ccl12	7.88	−10.94	
Plat	7.65	−24.16	
Mmp9	7.37	−15.05	
Hgf	7.14	−3.42	^*^
Fgf2	5.32	−27.25	
Fgf7	5.02	−28.67	
Fga	4.98	−2.46	^*^
Mmp7	4.87	−1.11	^*^
Col3a1	3.69	−20.75	
Tgfbr3	3.29	−8.56	From here excluded because DSS vs. UN < 3.5 folds
Cd40lg	3.28	1.65	
Mmp2	3.14	−5.55	
Tgfb1	3.12	−2.9	
Wisp1	3.1	−7.63	
Igf1	3.05	−5.33	
Il2	2.9	−4.12	
Col4a1	2.87	−9.7	
Fgf10	2.87	−16.81	
Cxcl11	2.84	−13.16	
Il4	2.77	−7	
F13a1	2.75	−6.89	
Itga4	2.55	−4.43	
Il6st	2.44	−4.13	
Itgav	2.43	−5.09	
Ctsk	2.33	−4.62	
Ctsg	2.21	−1.09	
Wnt5a	2.2	−6.11	
Plau	2.13	−4.31	
Angpt1	1.88	−3.51	
Col5a1	1.88	−5.81	
Plg	1.87	−1.14	
Col1a1	1.86	−2.32	
Itgb6	1.81	−3.41	
Stat3	1.79	−2.59	
Egfr	1.78	−4.44	
Itgb1	1.57	−4.24	
Col5a2	1.52	−9.03	
Itga5	1.52	−4.78	
Col1a2	1.44	−6.2	
Col5a3	1.39	−6.06	
Itga3	1.34	−1.48	
Itgb3	1.34	−3.19	
Itga2	1.17	−1.89	
Ctsl	1.16	−5.69	
F3	1.13	−3.34	
Itgb5	1.13	−2.25	
Hbegf	1.12	−6.42	
Mif	1.07	1.42	
Mmp1a	−1.01	−3.83	
Itga1	−1.04	−2.94	
Vegfa	−1.14	−1.39	
Ctnnb1	−1.22	−1.76	
Tagln	−1.23	−3.85	
Itga6	−1.25	−1.67	
Ctgf	−1.28	−11.17	
Rhoa	−1.29	−1.79	
Pdgfa	−1.35	−2.75	
Col14a1	−1.38	−1.9	
Pten	−1.4	−3.28	
Mapk3	−1.44	−1.06	
Mapk1	−1.44	−1.51	
Tgfa	−1.46	−1.52	
Cdh1	−1.56	−1.45	
Rac1	−1.66	−3.12	
Egf	−2.02	−2.22	
Csf2	−2.03	1.57	
Acta2	−2.13	−2.35	
Col4a3	−4.53	−1.52	
Actc1	−6.72	1.21	

*Inclusion criteria for the genes were the following: fold-regulation comparing DSS vs. UN > 3.5 folds; furthermore for those included fold-regulation comparing DSS+DPG vs. DSS > 3.5*.

*The genes were shown in order of decreasing up-regulation comparing DSS vs. UN*.

*The genes positive for the inclusion criteria were highlighted in gray*.

* *Genes excluded because DSS+DPG vs. DSS < 3.5 folds*.

**Figure 2:**
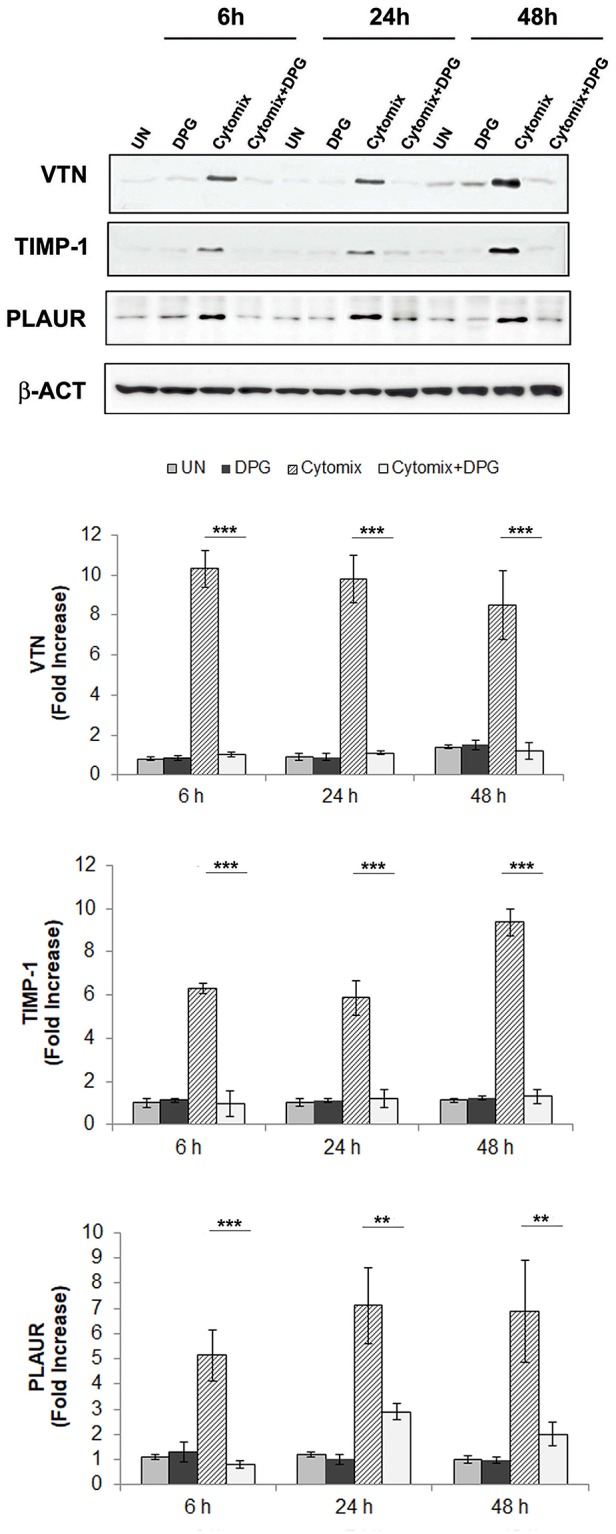
DPG reduced *in vitro* the expression of wound healing genes: VTN, TIMP-1, and PLAUR altered by inflammatory stimulus. HT-29 cells were exposed to cytomix or co-exposing to cytomix + DPG and protein expression of VTN, TIMP1, and PLAUR were analyzed by western blot. Bar chart represented fold increase (Densitometric Unit) vs. UN. The experiment was performed in triplicate and repeated 3 times. Statistical analysis: Mann-Whitney test, the comparison between Cytomix vs. Cytomix + DPG was reported. **p < 0.01, ***p < 0.001. UN, untreated cells; DPG, dipotassium glycyrrhizate.

This result was also confirmed *in vitro* by exposing Caco2 and HT-29 cells to pro-inflammatory cytokines (cytomix: TNF-α and IFN-γ) or co-exposing to cytomix + DPG and analyzing the protein expression of MMP9, VTN, COL3A1, TIMP1, and PLAUR by western blot. Results revealed a significant increase only of VTN, TIMP1 and PLAUR expression from 6 up to 24 and 48 h (*p* < 0.001) after cytomix exposure, however, co-exposure to DPG completely reverted to normal values (VTN and TIMP-1: *p* < 0.001; PLAUR: 6 h *p* < 0.001, 24–48 h *p* < 0.01) (Figure 2).

### Exposure to DPG Restores the Proper Structural Organization, Wound Repair Ability, and Functionality of Intestinal Epithelial Barrier Altered During Inflammation

**Figure 3:**
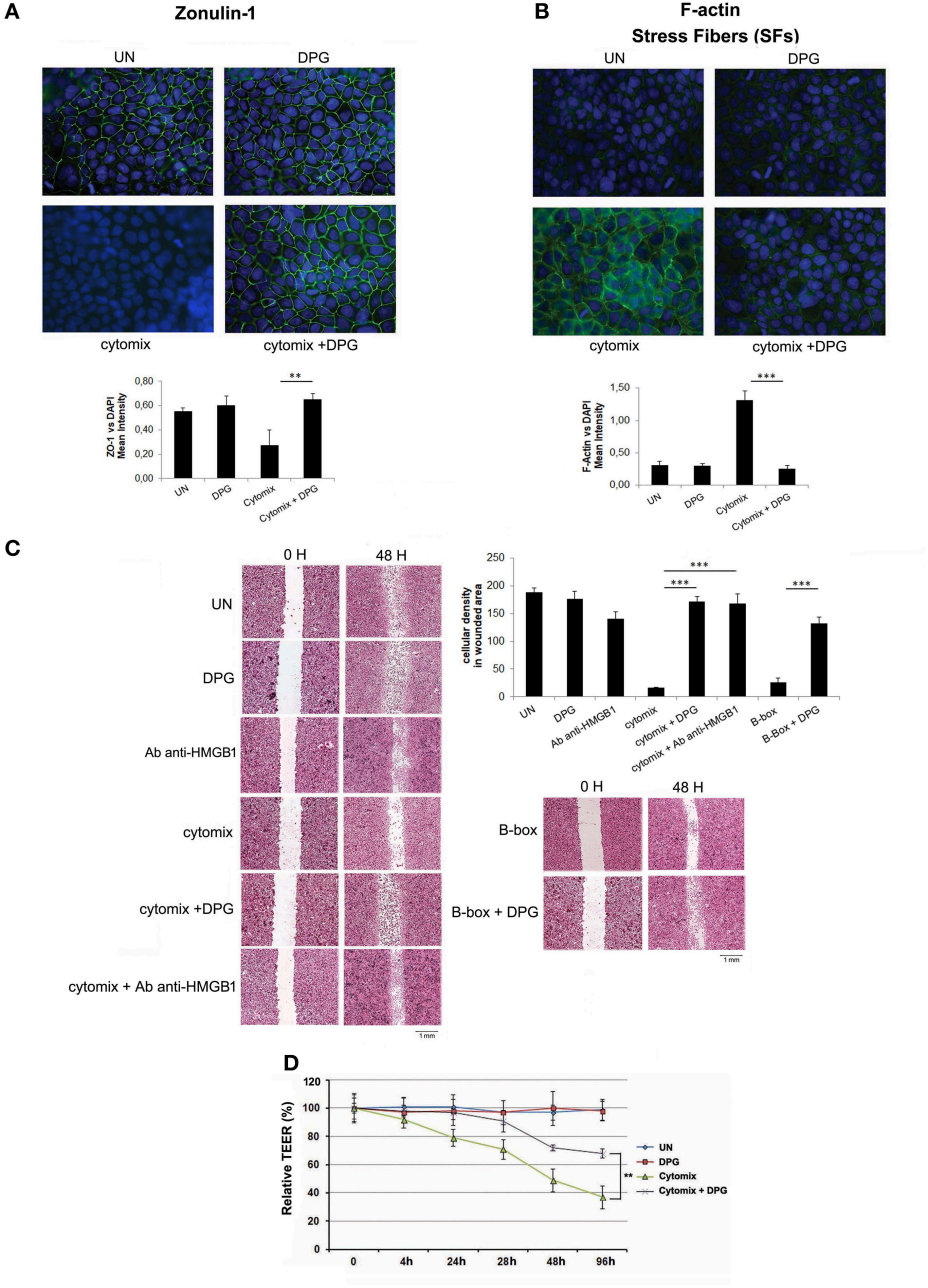
DPG improves *in vitro* epithelial barrier functions and morphology. Zonulin-1 and F-actin expression and localization were determined by immunofluorescence in Caco2 treated with Cytomix, DPG and Cytomix + DPG Results are presented as Mean intensity of ZO-1 and F-actin fluorescence, relative to DAPI ± S.D, respectively. Magnification 20X **(A,B)** Scratch test and bar chart of cellular density of wounded area in HT29 **(C)** and TEER values **(D)** indicated wound healing in Caco2 cells treated with Cytomix, DPG, and Cytomix + DPG. The experiment was performed in triplicate and repeated 3 times. Statistical analysis: Mann-Whitney test.**p < 0.01, ***p < 0.001. UN, untreated cells; DPG, dipotassium glycyrrhizate.

To evaluate the effect of DPG on epithelial barrier morphology, differentiated Caco2 and HT29 cells were exposed to cytomix or co-exposed to cytomix and DPG for 24 h. Cell-cell adhesion and intestinal epithelium organization were assessed by analyzing the expression levels of zonulin-1 and the formation of stress fibers (SFs), through immunofluorescence. All cells exposed to cytomix showed a strong reduction of zonulin-1 expression, with consequent loss of tight junction (TJ) adhesion, and an evident increase of SFs leading to a loss of proper tissue architecture. However, the exposure to DPG restored the original setting (ZO-1/DAPI fluorescence: UN: 0.55 ± 0.03; cytomix: 0.27 ± 0.13; cytomix + DPG: 0.65 ± 0.05); (F-actin/DAPI fluorescence: UN: 0.31 ± 0.05; cytomix: 1.30 ± 0.14; cytomix + DPG: 0.25 ± 0.05) (Figures 3A,B and Supplementary Figures 1A,B).

Furthermore, to assess whether DPG influences intestinal cell migration, a scratch test was performed. Since DPG is an inhibitor of HMGB1 we include a treatment with HMGB1 B box (B-box), which is the recombinant truncated form of the protein consisting of the pro-inflammatory component as well a treatment with anti-HMGB1 antibody, used to block HMGB1. Hence, confluent HT29 and Caco2, exposed to cytomix or to B-box in presence or absence of DPG or anti-HMGB1 antibody, were scratched with a 10 μl micropipette tip and the gap widths (1 mm at day 0) were measured after 24 and 48 h. Wound healing was estimated after 48 h as cellular density related to a fixed wounded area (1 mm^2^). Results showed that the cytomix exposure caused a delay in healing as compared to untreated cells (cellular density: cytomix 16.63 ± 0.95; UN 187.84 ± 8.25), however, co-treatment with DPG or with anti-HMGB1 antibody strongly reduced the delay, showing a complete repair after 48 h (cellular density: cytomix + DPG 171.15 ± 9.30; cytomix + anti-HMGB1 antibody 167.72 ± 17.78). Besides also B-box exposure caused a delay in healing strongly reduced by DPG (cellular density: B-box 26.39 ± 7.22; B-box + DPG 131.89 ± 11.53) (Figure 3C and Supplementary Figure 1C). Finally, the functionality of intestinal epithelial barrier was analyzed through the trans epithelial electrical resistance (TEER), a widely accepted quantitative technique to measure the integrity of tight junction (TJ) dynamics. TEER was applied to Caco2 cells that, when cultured until confluence, become a monolayer and differentiate spontaneously to enterocytes developing TJ on the apical side and getting excellent barrier functions (2 weeks culturing). After differentiation, cytomix or DPG+cytomix were given to the apical side of Caco2. Results showed that TEER values were significantly decreased in cells exposed to cytomix compared to physiological condition, indicating a loss of barrier functions (TEER value: 40% vs., untreated cells (TEER value: 100%) after 96 h of culture), while, DPG addition caused a sharp increase of TEER values, indicating a reliable recovery of barrier functions (TEER value: 70% after 96 h of culture) (Figure 3D).

### DPG Accelerates MH During the Recovery Phase Following the DSS-Induced Colitis in Mice by Differently Regulating Pro-inflammatory and Wound Healing Genes

**Figure 4:**
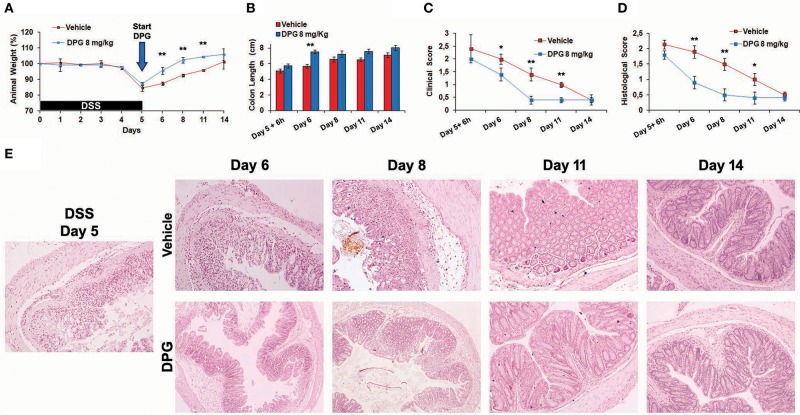
DPG accelerates mucosal healing during recovery phase of DSS-induced acute colitis. C57BL/6 mice were treated with 3% DSS for 5 days and then were untreated or treated with DPG (8 mg/kg) for successive 9 days. Following parameters were analyzed: **(A)** weight loss; **(B)** colon length; **(C)** clinical score (stool consistency, presence of blood in stool and general appearance); **(D)** histological score; **(E)** morphology of the colon evaluated through histological analysis (40x magnification in the image). Five animals per experimental group were used. Statistical analysis: Kruskal-Wallis Test with Dunn's Multiple Comparisons *post-Test*. *p < 0.05, **p < 0.01. UN, untreated animals; DSS, dextran sulfate sodium; DPG, dipotassium glycyrrhizate.

To investigate the effects of DPG on MH during the recovery phase following the induction of acute colitis, a time-course experiment was set up: mice were treated with DSS for 5 days and then with DPG (or a vehicle as placebo) for following 6-24-72-144-216 h (recovery time). Firstly, weight, colon length, clinical score and histological score were assessed. Results showed that mice treated with DPG had a faster recovery already visible at 24 h (day 6). Full recovery was obtained at 72 h (day 8) in DPG-treated mice and at 144 h (day 11) in vehicle-treated mice. In particular, at 24 h, DPG-treated mice recovered weight: 95 ± 2 vs. 87 ± 1.1% of vehicle-treated mice (*p* < 0.01) (Figure 4A); colon length: 7.5 ± 0.43 cm vs. 5.7 ± 0.33 cm of vehicle (*p* < 0.01) (Figure 4B); clinical score: 2.0 ± 0.2 vs. 1.4 ± 0.25 of vehicle (*p* ≤ 0.05) (Figure 4C); histological score: 0.9 ± 0.2 vs. 1.9 ± 0.3 of vehicle (*p* < 0.01) (Figures 4D,E).

**Figure 5:**
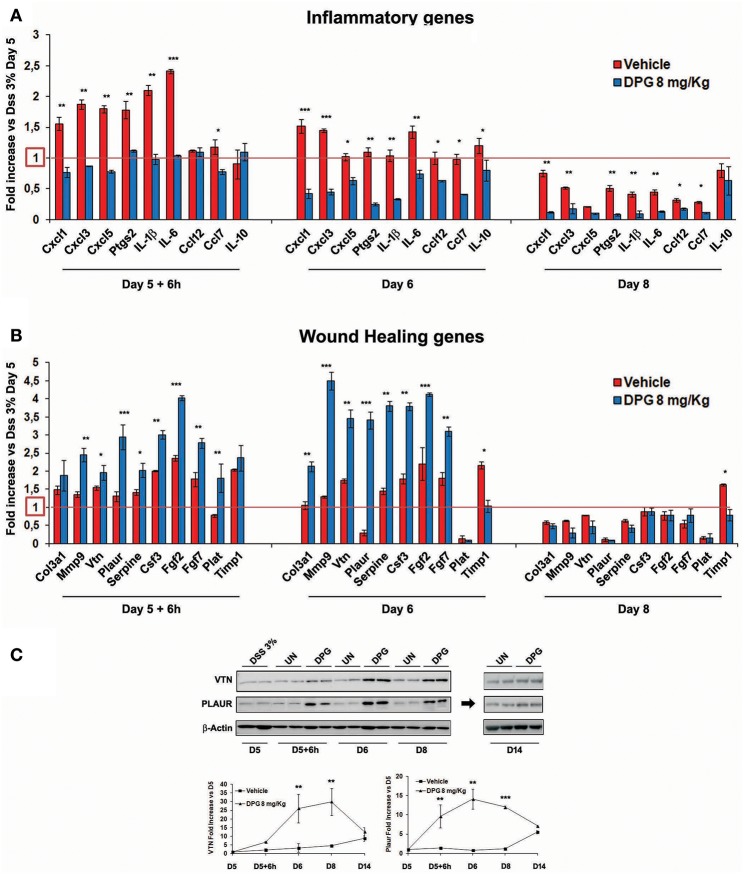
DPG improves mucosal healing *in vivo* by reducing pro-inflammatory genes and increasing wound healing genes. C57BL/6 mice were treated with DSS for 5 days and then were untreated or treated with DPG (8 mg/kg) for successive 9 days. During remission phase, gene expression of all **(A)** pro-inflammatory and **(B)** wound healing genes, previously identified, was analyzed by real-time PCR at the following time point: 5th day+6h, 6th day, and 8th day. Real-time PCR values are mean +/– St. dev. of 5 animals per experimental group and data represent the target gene expression normalized to the reference gene. The expression level after 5 days of DSS treatment was taken as 1 for each gene. **(C)** VTN and PLAUR were also analyzed by western blot. Upper panel: western blot; bottom panel: fold increase vs. day 5th calculated using optical densitometry. Five animals per experimental group were used. Statistical analysis: Kruskal-Wallis Test with Dunn's Multiple Comparisons *post-Test*.*p < 0.05, **p < 0.01, ***p < 0.001. UN, untreated animals; DSS, dextran sulfate sodium; DPG, dipotassium glycyrrhizate.

Furthermore, since results above show that the highest recovery rate occurred in the time window between 6 and 72 h (following the interruption of DSS), the mRNA levels of previously identified 19 genes were also analyzed in this time lapse. Results showed, post-DSS treatment, an increase of inflammatory molecules CXCL1, CXCL3, CXCL5, PTGS2, IL-1β, IL-6, CCL12, and CCL7 that were significantly reduced (24 h: *p* < 0.001: CXCL1, CXCL3; *p* < 0.01: PTGS2, IL-1β, IL-6; *p* < 0.05: CXCL5, CCL7, CCL12, IL-10) within the 72 h (back to control values) by DPG treatment (Figure 5A). Interestingly, DPG induced a significant increase (24 h: *p* < 0.001: MMP9, PLAUR, FGF2; *p* < 0.01: COL3A1, VTN, SERPINE, CSF3, FGF7; *p* < 0.05: TIMP1) of genes more directly involved in the wound healing, such as COL3A1, MMP9, VTN, PLAUR, SERPINE, CSF3, FGF2, FGF7, PLAT, and TIMP1, at earlier time (between 6 and 24 h) (Figure 5B). Noticeably, this result is not observed in untreated mice. Among all, the two most up-regulated genes by DPG, VTN and PLAUR, were also analyzed by western blot. Results confirmed that protein expression of both genes was significantly increased by DPG (VTN: 24–72 h *p* < 0.01; PLAUR: 6–24 h *p* < 0.01, 72 h *p* < 0.01) between 6 and 72 h comparing to untreated mice, but expression came back to control values at the end of treatment (day 14) (Figure 5C).

### The ECM Remodeling Proteins PLAUR and VTN Are Crucial to Achieve MH During DPG Treatment

**Figure 6:**
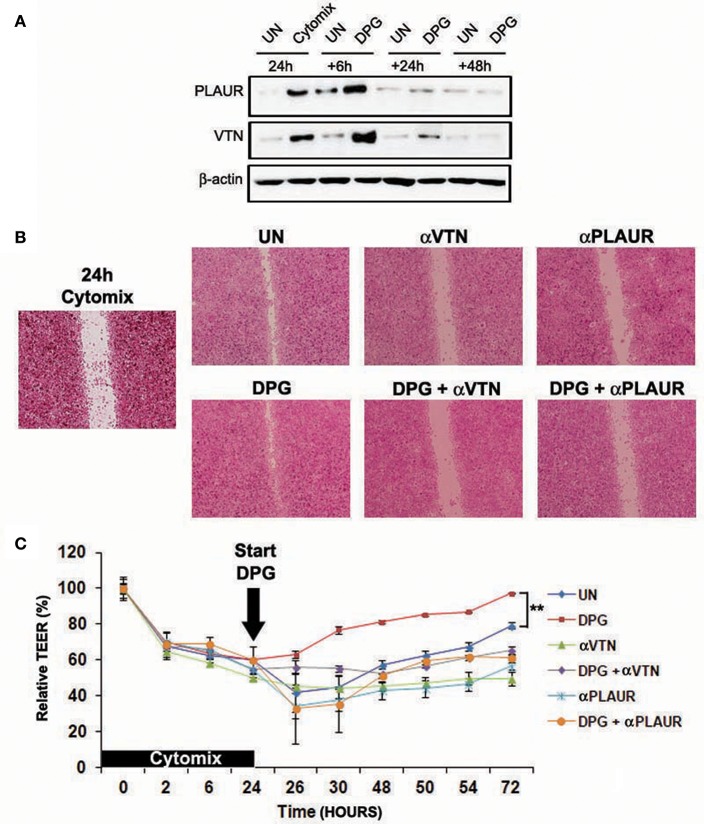
Proteins VTN and PLAUR are fundamental to achieve mucosal healing during DPG treatment *in vitro*. HT29 cells were pre-treated with cytomix for 24 h and then were exposed to DPG for 6, 24, and 48 h. **(A)** VTN and PLAUR expression was analyzed by western blot. In the same experimental condition as above HT29 or Caco2 were treated with DPG, with an anti-Plaur antibody or with the inhibitor of VTN, alone or in combination with DPG and **(B)** wound healing and **(C)** epithelial barrier integrity were evaluated by scratch test (HT29) and TEER (Caco2), respectively. The experiments were repeated in triplicate. Statistical analysis: Mann- Whitney test **p < 0.01. UN, untreated cells; DPG, dipotassium glycyrrhizate.

To investigate *in vitro* the role in MH of PLAUR, the most DPG-activated gene during the recovery phase from colitis at 6 h, as well as its ligand VTN, we observed the ability to repair and barrier function recovery of intestinal epithelial cells, exposed in time order to cytomix and DPG, in which VTN and PLAUR were alternately inhibited by specific antibody. Hence, Caco2 and HT29 cells were exposed to the cytomix for 24 h and then exposed to DPG for the following 6, 24, and 48 h. Firstly, results revealed that PLAUR and VTN expression significantly increased (*p* < 0.001) at 6 h following DPG and came back to normal values from 24 to 48 h (Figure 6A). Since this effect could indicate that these genes were required for an efficient MH, we carried out a scratch test and a TEER assay in inflamed epithelial cells in which VTN or PLAUR were inhibited by specific antibody. Thus, Caco2, and HT29 cells were treated with cytomix for 24 h, then cytomix was removed and DPG or DPG+antibody anti-VTN or DPG+antibody anti- PLAUR were added for the following 48 h for scratch test and up to 72 h for TEER assay. Results showed that the increase of TEER and wound closure rates induced by DPG was reverted by inhibition of VTN or PLAUR, suggesting that both genes are crucial in achieving MH during DPG treatment (Figures 6B,C).

## Discussion

Gut MH is a complex and dynamic process of replacing devitalized and missing cellular structures and tissue layers involving the coordinated activity of intestinal epithelial cells (20, 21). The ability of factors in modulating MH proteins and enhance intestinal repair mechanisms may form the basis of future approaches for treating diseases characterized by epithelial surface damage (22), since MH is emerging as a critical endpoint in clinical trials and practice (4). Indeed, it was shown that IBD patients who achieve and maintain MH have more favorable long-term outcomes compared to patients who do not get it (5). In the past two decades, anti-TNFα therapy was found to induce endoscopic remission and sustained mucosal healing (23), however, many patients (~40%) do not respond, lose their response during treatment, or develop complications due to side effects (24, 25). Recently, additional therapies were aimed to improve MH, such as vedolizumab (integrin blocker) and ustekinumab (anti-p40 subunit of IL-12/IL-23), mainly in patients unresponsive to anti-TNFα, but beneficial results are still variable and side effects persist (19, 26, 27).

In a previous study, the DPG was shown to importantly reduce the DSS-induced colitis in mice without collateral effects (17). In the present study, we firstly aimed to assess *in vivo* the ability of DPG to modulate a panel of MH genes. Accordingly, we induced the experimental acute colitis in mice and observed that DPG strongly down-regulated to control values 20 genes involved in different MH pathways (mainly represented by cytokine, chemokine and ECM genes), altered by inflammation. To deeply explore *in vitro* the effects of DPG on the gut barrier during the acute phase of inflammation, intestinal epithelial cells were co-exposed to inflammatory agents and DPG. Structural integrity of the intestinal epithelium was investigated by analyzing the modulated expression of the adhesion molecule, zonulin-1, and the dynamic alteration in actin fiber development. We found that DPG noticeably improved the expression levels of zonulin-1, strongly decreased by inflammation, and reduced the number of F-actin stress fibers resulting in response to inflammatory stimuli, altering the cell cytoskeleton organization. Moreover, since epithelial cell migration during healing represents an important mechanism to rescue the tissue integrity, we also looked at wound closure ability of intestinal cells and found that DPG significantly accelerated the healing rate during inflammation, improving epithelial barrier integrity but also its proper functioning, as seen by TEER assay. Of interest, rescue in tissue integrity due to DPG was observed when inflammation was trigger both by cytomix or B-box, suggesting that this effect is mediated by HMGB1 inhibition.

All these findings highlight the efficacy of DPG on improving wound healing during acute inflammation and, consequently, promoting the mucosal integrity, that is essential to preserve the epithelial functions. Indeed, patients with different gastrointestinal disorders, such as IBD, acute gastritis or pancreatitis, and celiac disease, are all phenotypically characterized by severely compromised epithelial barriers (22, 28) and re-establishment of barrier integrity is often associated with clinical remission and improved outcome (29).

In our study we found that the, DPG, an HMGB1 inhibitor, promote healing. Recently Tirone et al. (30), showed that HMGB1 promotes healing. Although these studies seem to disagree each other, the differences could be explained by the HMGB1 redox state. Indeed literature data reported that fully reduced HMGB1 (fr-HMGB1) acts as a chemo attractant for cells (31), whereas HMGB1 containing a disulfide bond (ds-HMGB1) is a pro-inflammatory molecule (32); further cysteine oxidation to sulfonates by reactive oxygen species abrogates both activities (33). To dissect the various activities of HMGB1, Tirone et al. created a mutant (3S) in which the cysteines are replaced with serines, which are resistant to sequential oxidation. Collectively, their results revealed that, after acute injury, muscle and liver regeneration were specifically promote by fr-HMGB1 and 3S. Furthermore, HMGB1 activities was analyzed during sterile injury. In our study we tested the effect of DPG, an HMGB1 inhibitor, on healing promotion in an inflammatory context. Probably, in our condition the prevalent form of HMGB1 should be the ds-HMGB1, with cytokine-inducing activity, and the DPG inhibiting it promotes healing.

Besides, we were interested to assess the effects of DPG during the recovery phase from colitis, as well. Thus, in a second set of experiments, mice were exposed to DSS and then let untreated (controls) or were treated with DPG for following 6, 24, 72, 144, or 216 h (recovery time). Intriguingly, we found that DPG-treated mice showed an evident improvement of body weight, colon length, clinical, and histological score as compared to controls. More interestingly, we observed since the earliest time (6 h) an opposite effect of DPG on MH gene expression: a significantly decrease of inflammatory genes (CXCL1, CXCL3, CXCL5, PTGS2, IL-1β, IL-6, CCL12, CCL7) and a simultaneous increase of ECM components/remodeling enzymes (COL3A1, MMP9, VTN, PLAUR, SERPINE, CSF3, FGF2, FGF7, PLAT, and TIMP1). These results clearly suggest that, within a narrow time window (between 6 and 24 h from the ending of DSS and the beginning of DPG treatment), while DPG is reducing inflammation, concurrently, is prompting the tissue rescue by implementing ECM remodeling genes. When inflammation is dampened (72 h following DPG treatment), ECM remodeling genes as well as inflammatory genes were wholly down-regulated. It is worth noting that it was possible to highlight the dual achievement of DPG only setting up a recovery phase experiment, indeed, we did not have this evidence in mice with the acute colitis.

These data drawn our attention on the role of ECM in MH. ECM is a highly dynamic structure present in all tissues continuously undergoing controlled remodeling that is critical for regulating the morphogenesis of different organs, including the intestine. Interestingly, ECM is established to be integral to wound healing, with a close relationship occurring between ECM deregulation and altered healing. In order to understand whether the ECM remodeling enzyme PLAUR, the most activated gene by DPG during the earlier recovery phase from colitis, as well as its ligand VTN, were critical for DPG-induced MH, we exposed to cytomix followed by DPG the intestinal cells, in which the two genes were alternately inhibited by specific antibodies. PLAUR, also known as urokinase-type plasminogen activator receptor (uPAR) is a part of the plasminogen activation system and is activated by several ligands as Serine protease urokinase-type plasminogen activator (uPA), VITRONECTIN (34) and inhibited by SERPINE1 (35).

Results showed that wound healing ability as well as epithelial barrier functions were importantly impaired by inhibition of both genes in inflamed cells then exposed to DPG. This evidence strongly suggests that VTN and PLAUR expression is mandatory to promote tissue repair even in cells in which MH is improved by DPG treatment. It is worth noting that under physiological conditions, PLAUR is thought to have fairly limited tissue expression. This receptor is expressed during ECM remodeling, for example in gestational tissues during embryo implantation and placental development (36) and in keratinocytes during epidermal wound healing (37). Stress, injury and inflammation also induce PLAUR expression. PLAUR-deficient mice exhibit pathological abnormalities related to PLAUR proteolytic function, including dermal fibrosis (38) and exacerbation of experimentally induced kidney fibrosis and nephropathy (39).

In conclusion, this study shows for the first time that: (1) DPG is able to modulate *in vivo* MH genes and restore *in vitro* the proper structural organization, wound repair ability and functionality of intestinal epithelial barrier during acute inflammation; (2) DPG increases ECM remodeling gene expression during the earlier recovery phase following the induction of colitis in mice; (3) the ECM genes, PLAUR and VTN, are pivotal to get MH in cells treated with DPG.

We are suggesting that the use of DPG would represent in the future an innovative and powerful tool for the treatment of human inflammatory complex disorders, including IBD.

## Ethics Statement

Experimental procedures were previously approved by the Ministry of Health and the study was carried out in accordance with the Italian regulations on animal welfare. The protocol was approved by the Committee on the Ethics of Animal Experiments of the Italian National Agency for New Technology, Energy and Sustainable Economic Development (ENEA-Permit Number: 1175/2016-PR).

## Author Contributions

LS and RV conceived and designed experiments. LS, FP, and ABM performed experiments and analyzed data. AN contributed to data analysis. EC and VC supervised *in vitro* analysis. SC and SI supervised the work. LS and RV wrote the manuscript.

### Conflict of Interest Statement

The authors declare that the research was conducted in the absence of any commercial or financial relationships that could be construed as a potential conflict of interest.

## Supplementary Material

Supplementary Material 1 Complete data set and False Discovery Rate of the microarray performed.

Supplementary Figure 1 DPG improves *in vitro* epithelial barrier functions and morphology. Zonulin-1 and F-actin expression and localization were determined by immunofluorescence in HT29. Treatment with DPG increases expression of **(A)** Zonulin-1 and reduces level of **(B)** F-actin altered by Cytomix. DPG significantly improved **(C)** wound healing in Caco2 cells, inhibited by Cytomix. UN, untreated cells; DPG, dipotassium glycyrrhizate.
