# Supplementary figures and images for: Dipotassium Glycyrrhizate Improves Intestinal Mucosal Healing by Modulating Extracellular Matrix Remodeling Genes and Restoring Epithelial Barrier Functions
Source: Front Immunol. 2019 Apr 26;10:939. doi: 10.3389/fimmu.2019.00939 (PMC6498413; doi:10.3389/fimmu.2019.00939)

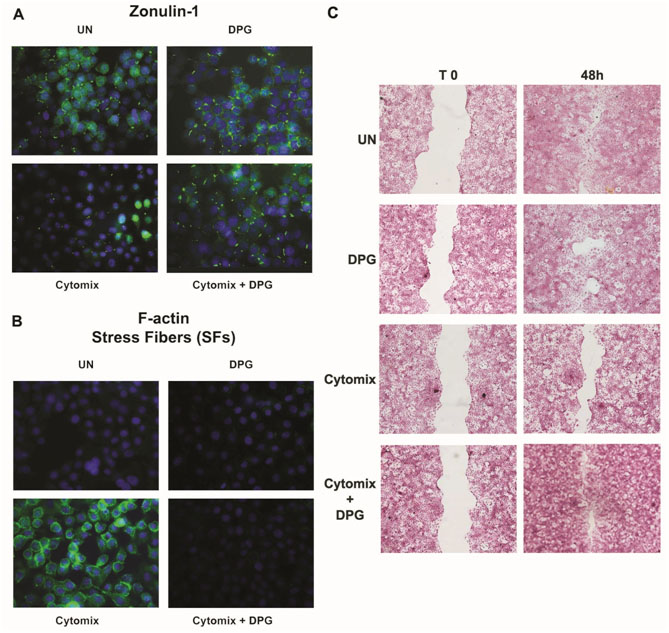

Supplement: Supplementary Figure 1 — DPG improves in vitro epithelial barrier functions and morphology. Zonulin-1 and F-actin expression and localization were determined by immunofluorescence in HT29. Treatment with DPG increases expression of (A) Zonulin-1 and reduces level of (B) F-actin altered by Cytomix. DPG significantly improved (C) wound healing in Caco2 cells, inhibited by Cytomix. UN, untreated cells; DPG, dipotassium glycyrrhizate. [file Image_1.JPEG]
